# Impact of ASHA Training on Active Case Detection of Visceral Leishmaniasis in Bihar, India

**DOI:** 10.1371/journal.pntd.0002774

**Published:** 2014-05-22

**Authors:** Vidya Nand Ravi Das, Ravindra Nath Pandey, Krishna Pandey, Varsha Singh, Vijay Kumar, Greg Matlashewski, Pradeep Das

**Affiliations:** 1 Rajendra Memorial Research Institute of Medical Sciences (ICMR), Patna, India; 2 Department of Microbiology and Immunology, McGill University, Montreal, Quebec, Canada; Common Heritage Foundation, Nigeria

## Abstract

**Background:**

One of the major challenges for management of visceral leishmaniasis (VL) is early diagnosis of cases to improve treatment outcome and reduce transmission. We have therefore investigated active case detection of VL with the help of accredited social health activists (ASHA). ASHAs are women who live in the community and receive performance-based incentives for overseeing maternal and other health-related issues in their village.

**Methods and Principal Finding:**

Through conducting interviews with 400 randomly selected ASHAs from four primary health care centers (PHCs), it was observed that their level of knowledge about visceral leishmaniasis (VL) regarding transmission, diagnosis, and treatment was limited. The baseline data indicated that less than 10% of VL cases seeking treatment at the PHCs were referred by ASHAs. To increase the knowledge and the referral rate of VL cases by ASHAs, training sessions were carried out during the monthly ASHA meetings at their respective PHCs. Following a single training session, the referral rate increased from less than 10% to over 27% and the overall knowledge about VL substantially improved. It was not possible, however, to demonstrate that ASHA training reduced the time that individuals had fever before treatment at the PHC.

**Conclusions:**

Training ASHAs to identify VL cases in villages for early diagnosis and treatment at the local PHC is feasible and should be undertaken routinely to improve knowledge about VL.

## Author Summary

Visceral leishmaniasis (VL) is a potentially deadly parasitic disease that affects over 20,000 people in Bihar, India annually. Accredited social health activists (ASHA) are women who live in the community and render their services towards maternal and other health concerns in the villages where VL is endemic. One of the major problems is that VL cases remain in the village without seeking treatment and, during this period, continue to transmit the disease. To address this problem, we have investigated the possibility of training ASHAs to identify potential VL cases and to send them for treatment at the local primary health care centers (PHCs). We demonstrate that ASHA knowledge about VL increased significantly with training and this resulted in increased recruitment of patients for diagnosis and treatment to the local PHCs. These observations demonstrate that training of ASHAs should be conducted routinely to support the elimination of VL.

## Introduction

There are estimated 200,000 to 400,000 new cases of visceral leishmaniasis (VL), also known as kala-azar [1] every year worldwide. Bihar state in India contributes the majority of the disease burden of VL which is most prevalent in poorest populations [1], [2]. The major risk factor for infection is living in the same household as someone with VL patients therefore it is important to identify and treat people as soon as possible [3], [4]. Serological diagnosis with the rk39 rapid diagnostic test (RDT) can be performed on a single drop of blood on individuals with prolonged fever and splenomegaly [4], [5]. Early diagnosis and complete treatment with new drugs such as oral miltefosine, paromomycin and liposomal amphotericin B used alone or in combination with each other can contribute to the elimination of VL [6]–[9].

Notwithstanding how effective VL point of care the diagnosis and treatments are, VL will continue to be transmitted and remain embedded in the community if VL cases do not seek early treatment. Awareness remains a major challenge in the most endemic districts of Bihar when considering the scale of the problem which involves thousands of villages [10], [11]. Conducting one day fever camps is an effective approach to identify VL cases in highly endemic villages but requires significant resources when performed on large scale [11], [12]. We have therefore considered a different approach for case detection including enlisting the involvement of community health care workers, also known as **a**ccredited **s**ocial **h**ealth **a**ctivists (ASHA) to help identify potential VL cases in the endemic villages.

One of the key strategies under the National Rural Health Mission (NRHM) in India is to have one ASHA for every 1000 villagers in rural populations. There are over 82,000 ASHAs for a population of 100 million people in the state of Bihar (Source: State Health Society, Bihar). Selected from the village, ASHAs are women between 25 and 45 years with a minimum level of formal education who are trained to work as an interface between the villages and public health system and receive performance based incentives for promoting the different health care programs [13], [14]. ASHAs are provided with training to acquire knowledge, skill and confidence for performing roles in immunization, referral and escort services for family planning, antenatal care and child health with the aim to reduce infant and maternal mortality. Some ASHAs have been trained to bring other ailing rural populations to the PHCs for diagnosis and treatment to reduce transmission of disease including for example HIV infections [15]. Recently it has been reported that ASHAs in Bihar were generally aware of VL but that their knowledge of treatment was limited and few were involved in VL control [16].

The National Vector Borne Disease Control Program of India has recently recognized the potential for training ASHAs to identify potential VL cases in the villages and has provided an incentive of 200 rupees for every case registered in PHCs and ensuring complete treatment. The purpose of this study was to determine whether targeted training of ASHAs can increase their knowledge of VL and whether this will result in increased recruitment of VL patients to the PHC for diagnosis and treatment.

## Methods

### Study Design

Two districts, Muzaffarpur and Saran were selected on the basis of high VL and post kala-azar dermal leishmaniasis endemicity. The distance between these districts was about 80 kilometers. One highly endemic PHC from each of the two districts was selected as intervention PHCs (Paroo and Marhoura) where the ASHAs received training. Similarly, one highly endemic PHC from each of two districts was selected as control PHCs (Sahebganj and Baniyapur) where training was not conducted. The total population of the four PHCs is nearly two million. Approximately five hundred ASHAs (in batches of approximately hundred) from two intervention PHCs were trained on VL and PKDL case identification and on referral to PHCs for diagnosis and treatment. Training was provided during ASHA monthly meeting days which was cost effective in moving large numbers of ASHAs from village to PHC for training. Training was provided by experts from the research team using standard power point presentation, photographs and discussion. The importance of active case detection, early diagnosis and complete treatment in control of VL transmission was explained to the ASHAs. The knowledge of vector and its control by insecticide spraying was also explained to ASHAs extending their role and cooperation during spraying activity in their villages. Trained ASHAs were given a booklet on Kala-azar written in Hindi, register, carry bag, referral slip and a poster to place in their village describing what to do if someone has VL and PKDL symptoms.

### Ethics Statement

The study was approved by the ethics committee of the Rajendra Memorial Research Institute of Medical Sciences, Patna, India. Subjects participated in the research after a written informed consent.

### Data Collection and Analysis

Data was collected by field assistants trained by faculty of the Rajendra Memorial Research Institute of Medical Sciences (RMRIMS), Patna and support staff under direct supervision of the investigator team. Two trained field assistants were deployed in each of the 4 PHCs in the study. Data on VL cases was collected for both intervention and control PHCs before ASHA training (2011) and after ASHA training (2012). Structured questionnaires prepared and pretested by faculty of RMRIMS were used for data collection from VL and PKDL cases treated in the 4 PHCs during 2011 and 2012. Data was collected on treatment, compliance, duration of fever, first point of contact for health care after onset of symptoms and by whom the patients were referred for treatment. After training of ASHAs in March 2012, data was again collected for VL and PKDL positive cases for the year 2012 (April 2012 to December 2012). To assess ASHA knowledge on different aspects of VL and PKDL and vector control after training in March 2012, a questionnaire was administered to randomly selected 100 ASHAs from all 4 PHCs (trained vs. non-trained) from October to November 2012 and their knowledge were evaluated. This did not affect the working of the ASHAs in their village. Data entry was done in EpiInfo version 3.5.1, software specifically designed for the study. Data analysis was done in Graph Pad, online Statistical Software.

## Results

**Figure 1:**
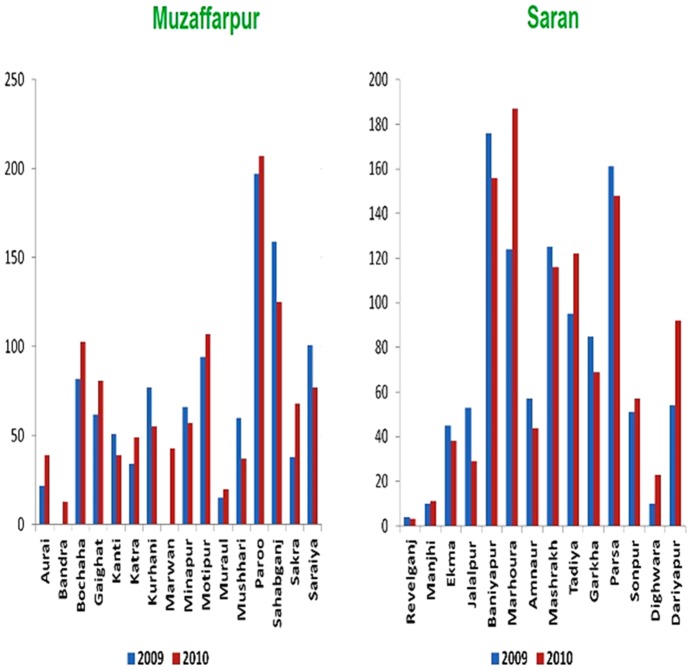
Number of visceral leishmaniasis cases in 2009 and 2010 in all the PHCs of Muzaffarpur and Saran disctricts indicating the highest number of cases in Paroo and Sahebganj of Muzaffarpur district and Baniyapur and Marhoura of Saran district.

Initially we determined which PHCs had the highest number of VL cases in 2009 and 2010 in Muzaffarpur and Saran districts of Bihar State so that these could be selected for this study. As shown in Figure 1, using State government medical records, the highest number of treated cases was in Paroo and Sahebganj PHCs in Muzaffarpur district and Baniyapur and Marhoura PHCs in the Saran district. We therefore selected Paroo, Sahebganj, Baniyapur and Marhoura PHCs for this study.

Using medical records from the PHCs, it was possible to obtain relevant information on the treated VL patients including where they lived, when they were treated and what they were treated with. Field assistants traveled into the community to locate the VL cases and obtained information including how long the VL cases were ill before seeking treatment, why they sought treatment at the PHC, and whether they were cured of symptoms. Once the base-line information was obtained, ASHAs received training about VL and PKDL through a power point presentation and discussion at the Paroo and Marhoura PHCs. Training was conducted during the routine monthly meetings where typically 100–200 ASHAs attended. The control PHCs were Sahebganj and Baniyapur where baseline information was also gathered but the ASHAs were not trained. Approximately 6 months after training, ASHAs from the intervention PHCs (Paroo, Marhoura) and control PHCs (Sahebganj, Baniyapur) were interviewed to determine their level of knowledge about VL. As shown in Table 1, there was a significant increase in the basic knowledge about transmission, diagnosis and treatment in the group of ASHAs from the intervention PHCs compared to the ASHAs from the control PHCs. This demonstrates that the training session did improve knowledge about VL. It is particularly noteworthy that considerable more ASHAs in the trained group were aware that treatment with miltefosine required 4 weeks and this should help with compliance in the future.

**Table 1:** ASHA knowledge in Control (untrained) and Intervention (trained) PHC.

Indicator	Control PHC (Base line)	Intervention PHC (Post-Training)
No. of ASHA Interviewed	200	200
Knew about diagnosis with rK39	21 (10%)	112 (56%)
Knew about treatment duration with Miltefosine	37 (18%)	160 (80%)
Knew about transmission by infected sand fly	116 (58%)	191 (95%)
Knew about symptoms of VL	126 (63%)	174 (87%)

Difference between intervention and control groups for all indicators was p<0.05.

Table 2 shows the results for VL case referrals by the ASHAs in 2011 (period prior to training) and 2012 (period following training). In the intervention PHCs Paroo and Marhoura, referrals by ASHAS were 9.8% and 4.0% respectively in 2011 and this increased following training to 28.3% and 27.5% in 2012. In the control PHCs Sahebganj and Baniyapur, referrals were 4.5% and 6.2% in 2011 respectively and 26.6% and 9% in 2012. The increase in referrals from 4.5% to 26.5% in the control PHCs, Sahebganj was unexpected. We determined that this was due to a single village (Tarawa) where one ASHA referred 14 VL cases making up the majority of the total cases referred to the Sahebganj PHC. The ASHA in Tarawa had become informed about VL from the field assistants when baseline information on previous VL cases was collected from her village in 2011. Subsequently in 2012, there were 14 VL cases in Tarawa and the ASHA referred all of these cases to the Sahebganj PHC. If these 14 cases were not included in the analysis (shown in brackets in Table 2), then the percentage of cases referred to this control PHC in 2012 would be 10%.

**Table 2:** VL cases referred by ASHAs before and after training.

	PHC	2011: Before ASHA training			2012: After ASHA training		
		1Total cases	ASHA referral	Referral %	1Total cases	ASHA referral	Referral %
Intervention (Trained)	Paroo	153	15	9.80	67	19	28.35
	Marhoura	149	6	4.03	69	19	27.54
Control (Untrained)	Sahebganj	133	6	4.51	79 (65)^2^	21(7)	26.58 (10.77)^2^
	Baniyapur	177	11	6.21	89	8	8.99

1 The total number of cases in 2011 is more than that in 2012. This was because in 2011, cases from 12 months were followed-up, whereas in 2012, cases from only 6 months were followed-up. The increased recruitment rate between 2011 and 2012 observed in the intervention PHCs, Paroo and Marhoura was statistically significant (p<0.05).

2 Numbers in brackets represent the number of cases after removing the 14 cases that were referred by the ASHA from Tarawa village that received knowledge about VL from the study team in 2011. The increased recruitment rate at Sahebganj PHC between 2011 and 2012 was significant (p<0.05), but was not significant after removal of the 14 cases from Tarawa (p = 0.12).

We also attempted to determine the time that the VL cases had fever before seeking treatment at the PHC and whether this interval was shortened following ASHA training. The recorded mean duration of fever however did not change between 2011 (before training) and 2012 (after training) and varied from 5–7 weeks as shown in Table 3. This could indicate that ASHA training does not reduce the time before patients seek treatment. Alternatively, the reason there was no change may be due to the difficulty to accurately determining the length of time that fever was present through the interview process.

**Table 3:** Duration of fever before seeking treatment at the PHC.

	PHC	Mean duration of fever (in days)	
		2011: Before ASHA training	2012: After ASHA training
Intervention (Trained)	Paroo	37	36
	Marhoura	36	32
Control (Untrained)	Sahebganj	50	48
	Baniyapur	44	43

## Discussion

This study was initiated to address the challenges imposed by scaling up active case detection of VL cases when the target region involves thousands of villages in multiple districts and resources are limited. We therefore considered interventions that use existing resources. ASHAs live in the endemic villages, are aware of community health issues and are accessible as a large group during monthly meeting sessions at their local PHCs. ASHAs have already been involved in improving different health care programmes under NRHM [14], [15] and have been reported to have a basic knowledge of VL [16]. Our results support the conclusion of the previous study and extend this by showing that ASHA knowledge of VL can be substantially augmented by additional training at their local PHCs.

Several key observations were made in this study. First, although of 50% of the ASHAs in this study had a basic knowledge of VL symptoms and transmission, less than 20% of the ASHAs were familiar with diagnosis and current treatment of VL despite being in the most highly endemic districts. This knowledge increased dramatically at 6 months following the training when 80% of ASHAs were familiar with the time necessary for treatment with miltefosine. This could be useful to increase treatment compliance. Second, it was possible to more than double the percentage of patients recruited to the government PHCs following only one ASHA training session during one of their monthly meetings at the PHC. We expect this would increase if the training were performed at least 2 times per year.

In addition to the training sessions, approximately 500 ASHAs were also provided with a poster to be placed in a prominent place in 500 villages to inform people about VL and PKDL symptoms and that diagnosis and treatment is free of cost at the local PHC. We believe that display of posters in 500 villages helped to create awareness of VL and PKDL reaching about one million rural populations in the highly endemic area. We are currently in the process of conducting training for ASHAs in control PHCs with the distribution of posters for wider dissemination of knowledge regarding VL.

There were however several drawbacks of this study. First, it was difficult to have an unbiased control group because ASHAs and villagers became more knowledgeable of VL during the collection of baseline information in the control villages. As described in the results section, one ASHA from a highly endemic control village (Tarawa) had discussed the study with the field assistants as they interviewed previous VL cases. As a result, this ASHA became knowledgeable about VL and sent 14 cases to the Sahebganj PHC. Although this compromised the control group, it did demonstrate that the ASHA was attentive to health related issues in her village and responded appropriately.

Another drawback was the difficulty to confidently determine how long patients had fever prior to seeking treatment at the PHC. Although low-grade fever may be present, this is generally not considered an illness in these villages making it difficult to accurately measure time of fever. Furthermore, many VL cases had initially sought treatment from local quacks which further complicated the ability to accurately determine the time of fever before seeking attention at the PHC. Better refinement of the questionnaire could help. For example, asking the patient to describe their symptoms and then asking how long they had those symptoms before going to the PHC may be more effective way in determining the duration of illness.

Under the current government supported VL elimination program, ASHAs should be paid 200 rupees for every VL case they identify. The current cash incentive scheme is not functioning properly because of poor financial management at district and sub-district levels. For the purpose of this study, ASHAs were paid from study funds but clearly the issue of payment must be corrected otherwise the training program will lose credibility. Reducing the time a VL case remains in the village will reduce the time of disease transmission and reduce cases. Therefore the reimbursement of ASHAs would represent a significant cost saving to the government.

Taken together, the observations from this study argue that training ASHAs at the PHCs is feasible and should be undertaken to support the active case detection of VL cases in endemic districts of Bihar. Furthermore, this would also recruit non-VL febrile cases to the PHCs to identify and treat other infections in the area.
